# Stargazin Dephosphorylation Mediates Homeostatic Synaptic Downscaling of Excitatory Synapses

**DOI:** 10.3389/fnmol.2018.00328

**Published:** 2018-09-13

**Authors:** Susana R. Louros, Gladys L. Caldeira, Ana Luísa Carvalho

**Affiliations:** ^1^CNC-Center for Neuroscience and Cell Biology, University of Coimbra, Coimbra, Portugal; ^2^Department of Life Sciences, University of Coimbra, Coimbra, Portugal

**Keywords:** homeostatic plasticity, stargazin, AMPA receptors, synaptic downscaling, membrane trafficking

## Abstract

Synaptic scaling is a form of homeostatic plasticity that is critical for maintaining neuronal activity within a dynamic range, and which alters synaptic strength through changes in postsynaptic AMPA-type glutamate receptors. Homeostatic scaling down of excitatory synapses has been shown to occur during sleep, and to contribute to synapse remodeling and memory consolidation, but the underlying mechanisms are only partially known. Here, we report that synaptic downscaling in cortical neurons is accompanied by dephosphorylation of the transmembrane AMPA receptor regulatory protein stargazin, and by an increase in its cell surface mobility. The changes in stargazin surface diffusion were paralleled by an increase in the mobility of GluA1-containing AMPA receptors at synaptic sites. In addition, stargazin dephosphorylation was required for the downregulation of surface levels of GluA1-containing AMPA receptors promoted by chronic elevation of neuronal activity, specifically by mediating the interaction with the adaptor proteins AP-2 and AP-3A. Disruption of the stargazin-AP-3A interaction was sufficient to prevent the decrease in cell surface GluA1-AMPA receptor levels associated with chronically enhanced synaptic activity, suggesting that scaling down is accomplished through decreased AMPA receptor recycling and enhanced lysosomal degradation. Thus, synaptic downscaling is associated with both increased stargazin and AMPA receptor cell surface diffusion, as well as with stargazin-mediated AMPA receptor endocytosis and lysosomal degradation.

## Introduction

Homeostatic synaptic scaling protects information coding in the brain by allowing neurons to keep neuronal activity within a physiological range while constantly facing destabilizing changes in synaptic strength. During homeostatic scaling, neurons adjust the strength of synapses proportionally, thereby maintaining the differences in synaptic strength that store information in neuronal circuits. Synaptic scaling was initially described in cultured neocortical neurons, after pharmacological manipulation of activity elicited compensatory and bidirectional changes in the strength of glutamatergic synapses through AMPA receptor accumulation (Turrigiano et al., 1998). A physiological function has been demonstrated for homeostatic scaling in the visual system in response to sensory experience (Desai et al., 2002; Goel et al., 2006; Hooks and Chen, 2008; Hengen et al., 2013), and scaling down has been shown to occur during sleep, and to contribute to synapse remodeling (Diering et al., 2017; de Vivo et al., 2017) and to the consolidation of contextual memory (Diering et al., 2017). Synaptic downscaling of excitatory synapses occurs upon prolonged elevation of neuronal activity, and leads to synapse weakening through the synaptic removal of AMPA receptors, but the molecular mechanisms that underlie synaptic downscaling are poorly characterized. Alterations in the signaling mediated by group I metabotropic glutamate receptors (Hu et al., 2010; Diering et al., 2017), by PKA (Diering et al., 2014), phosphatase PP1 activation (Siddoway et al., 2013) and secreted semaphoring 3F (Wang et al., 2017) contribute to synaptic downscaling. Moreover, ubiquitination of AMPA receptor subunits and their proteasomal and/or lysosomal degradation are implicated (Hou et al., 2011; Scudder et al., 2014), and the synaptic removal of the scaffold protein PSD-95 is required for scaling down (Sun and Turrigiano, 2011), through a mechanism that involves Ca^2+^/calmodulin binding to the N-terminus of PSD-95 (Zhang et al., 2014; Chowdhury et al., 2018).

AMPA receptors are anchored at postsynaptic sites through the binding of auxiliary proteins, such as transmembrane AMPA receptor regulatory proteins (TARPs), to PSD-95. One such protein is stargazin, which binds to AMPA receptors to regulate both AMPA receptor trafficking and channel gating (Nicoll et al., 2006). Stargazin leads to the diffusional trapping of AMPA receptors at synapses (Bats et al., 2007; Opazo et al., 2010), and its phosphorylation is required for dissociation from negatively charged lipid bilayers and interaction with PSD-95 (Sumioka et al., 2010), leading to interaction with deep domains of PSD-95 (Hafner et al., 2015) and synaptic immobilization of AMPA receptors (Opazo et al., 2010). In addition, it is required for long-term potentiation (Tomita et al., 2005) and for synaptic upscaling (Louros et al., 2014). Activity-dependent stargazin dephosphorylation, on the other hand, plays a role in long-term depression (Tomita et al., 2005; Matsuda et al., 2013), through the regulation of AMPA receptor endocytosis and lysosomal degradation (Matsuda et al., 2013). Despite the crucial role of stargazin in controlling multiple steps of AMPA receptor trafficking, gating and pharmacology, it is not known whether stargazin is implicated in synaptic downscaling.

In the current study, we addressed whether stargazin is implicated in synaptic scaling upon prolonged elevation of neuronal activity. We modeled synaptic downscaling *in vitro*, by chronically inhibiting GABA_A_ receptor activity in cultured cortical neurons, a paradigm that has been shown to elicit a decrease in the amplitude of AMPA receptor-mediated miniature postsynaptic currents (mEPSCs; Turrigiano et al., 1998), and a reduction in the cell surface levels of AMPA receptors (Chowdhury et al., 2018). We quantified stargazin phosphorylation levels and cell surface diffusion, and evaluated whether the phosphorylation-dependent stargazin role in mediating AMPA receptor endocytosis and degradation is implicated in homeostatic downscaling. We found that stargazin dephosphorylation during scaling down results in an increase in its cell surface diffusion, which is accompanied by an increase in the mobility of GluA1-containing AMPA receptors specifically at synaptic sites. In addition, we found that stargazin phosphonull mutations are permissive to scaling down, while the stargazin phosphomimetic mutant abolishes synaptic downscaling. Finally, inhibition of the dephosphorylation-dependent interactions of stargazin with the adaptor proteins AP-2 or AP-3A, which disrupt AMPA receptor endocytosis and late endosomal/lysosomal trafficking of AMPA receptors, blocked synaptic downscaling, suggesting that stargazin dephosphorylation mediates downscaling by promoting AMPA receptor endocytosis and lysosomal degradation.

## Materials and Methods

### Cortical Neuron Cultures

Primary cultures of rat cortical neurons were prepared from the cortices of E18 Wistar rat embryos, as previously described (Louros et al., 2014). This procedure was reviewed and approved by the Portuguese National Authority for Animal Health (DGAV). Dissected cortices were treated with trypsin (0.06%, 15 min, 37°C; GIBCO Invitrogen), in Ca^2+^- and Mg^2+^-free Hank’s balanced salt solution (HBSS in mM: 5.36 KCl, 0.44 KH_2_PO_4_, 137 NaCl, 4.16 NaHCO_3_, 0.34 Na_2_HPO_4_.2H_2_O, 5 glucose, 1 sodium pyruvate, 10 HEPES and 0.001% phenol red). Cortical cells were washed with HBSS six times. Cells were plated in neuronal plating medium (MEM supplemented with 10% horse serum, 0.6% glucose and 1 mM pyruvic acid) in 6-well plates (9 × 10^5^ cells/well), coated with poly-D-lysine (0.1 mg/ml). After 2–4 h the medium was replaced by fresh Neurobasal medium supplemented with SM1 (1:50), 0.5 mM glutamine and 0.12 mg/ml gentamycin.

For imaging purposes, low-density cortical cells were plated at a final density of 3 × 10^5^ cells/dish on poly-D-lysine-coated coverslips, in 60 mm culture dishes, in neuronal plating medium. After 2–3 h, coverslips were flipped over an astroglial feeder layer, in Neurobasal medium supplemented with SM1. To prevent the overgrowth of the glia, neuron cultures were treated with 5 μM cytosine arabinoside after 3 days *in vitro* (DIV). Cultures were fed twice a week and maintained in Neurobasal medium supplemented with SM1 supplement, in a humidified incubator of 5% CO_2_, at 37°C. The astroglial feeder layer was prepared from the cortices of E18 Wistar rat embryos, which were chemically dissociated with trypsin (0.06%, 15 min, 37°C; GIBCO Invitrogen) in HBSS. Dissociated cells were plated in 75 cm^2^ culture flasks (Corning) with Glial medium (MEM supplemented with 10% horse serum, 0.6% glucose and 1% Penicillin/Streptomycin) which was changed in the day following the plating and every 3 days afterwards. Once reaching confluence (average time ~15 days), cells were treated with trypsin, plated in 60 mm culture dishes and maintained for at least 7 days in a humidified incubator of 5% CO_2_, at 37°C, before use.

To induce synaptic scaling, neurons were treated with 100 μM picrotoxin (PTX) for a maximum of 24 h at DIV14–15. DIV15–16 the neurons were lysed with TEEN buffer (25 mM Tris-Cl, pH 7.4, 1 mM EDTA, 1 mM EGTA, 150 mM NaCl and 1% Triton X-100) supplemented with 50 mM sodium fluoride (NaF), 1.5 mM sodium orthovanadate (Na_3_VO_4_) and a cocktail of protease inhibitors. For the λ-phosphatase (λ-PPase) assay, no phosphatase inhibitors were added to the lysis buffer.

### DNA Constructs

Stargazin phosphomutants, S9A and S9D, were a kind gift of Dr. Susumu Tomita (University of Yale, New Haven, CT, USA). Wild-type stargazin (WT), stagazin-ΔAP2 and stargazin-ΔAP3 mutants were obtained from Dr. Michisuke Yuzaki (Keio University, Japan). Stargazin-HA and Homer1-GFP were a kind gift from Dr. Daniel Choquet (University of Bordeaux, France). All constructs were verified by DNA sequencing.

### Neuron Transfection

Constructs were recombinantly expressed in primary cultures of cortical neurons using the calcium phosphate transfection protocol (adapted from Jiang et al., 2004). This protocol is a low-efficiency transfection method that allows 20%–50% of transfected neurons. Briefly, a CaCl_2_ solution (2.5 M in 10 mM HEPES) was combined with plasmid DNA and then added to an equivalent volume of HEPES buffered transfection solution (274 mM NaCl, 10 mM KCl, 1.4 mM Na_2_HPO_4_, 11 mM dextrose, and 42 mM HEPES, pH 7.2). After 30 min incubation the precipitated DNA was added to the coverslips and the cultures were incubated for 1.5 h in the presence of kynurenic acid (2 mM). Coverslips were then transferred back into the original astroglial plate and the plasmids were allowed to express during the indicated times.

### Immunocytochemistry, Culture Imaging and Quantitative Fluorescence Analysis

Surface staining of GluA1 was performed by incubating the cells with an antibody against an extracellular epitope of GluA1 (antibody was kindly provided by Dr. Andrew Irving, Santos et al., 2012), in culture medium, for 10 min, at room temperature, before fixation. Neurons were fixed for 15 min in 4% sucrose/4%paraformaldehyde in PBS at room temperature, and permeabilized with PBS + 0.25% Triton X-100 for 5 min, at 4°C. The neurons were then incubated in 10% BSA in PBS for 30 min at 37°C to block nonspecific staining, and incubated with the indicated antibodies diluted in 3% BSA in PBS (2 h, 37°C or overnight, 4°C). Imaging was performed on a Zeiss Axiovert 200M microscope, using a 63×-1.4 NA oil objective. For quantification, sets of cells were cultured and stained simultaneously, and imaged using the same exact settings. Images were quantified using image analysis software (ImageJ). The region of interest was randomly selected and the dendritic length was measured using the MAP2 (Abcam, ab5392) staining. For quantifying the GluA1 signal, fields for imaging were chosen by the GFP channel, for the presence of transfected, GFP positive, neurons. Surface GluA1 and total stargazin (Millipore, mab 9876) digital images were thresholded such that recognizable clusters were included in the analysis. The ImageJ function “analyze particles” allowed us to calculate cluster intensity for the selected region. The synaptic GluA1 and stargazin clusters were selected by their overlap with thresholded PSD95 (Thermo Scientific Pierce, PIEAMA1045) or Vglut1 (Millipore, AB5905) signal. The analysis was performed blind to condition.

### SDS-PAGE and Western Blot

Samples were processed by SDS-PAGE in 11% polyacrylamide gels. Proteins were transferred overnight at 40 V to a PVDF membrane (Millipore). After blocking with 5% blocking solution (GE Healthcare), in 0.1% Tween-20 supplemented TBS (20 mM Tris, 137 mM NaCl, pH 7.6; TBS-T), membranes were incubated with an anti-stargazin antibody (Millipore, mab9876) and anti-α-tubulin (Abcam, ab7291), overnight at 4°C or 1 h at RT, respectively. Following three 15 min washes with TBS-T, membranes were incubated with alkaline phosphatase-conjugated secondary antibody (in 5% blocking solution in TBS-T) for 45 min at RT and then washed three times with TBS-T. The membranes were developed with the alkaline phosphatase substrate ECF, and the fluorescent signal was acquired in a Storm 860 Gel and Blot system (GE Healthcare).

### Lambda Phosphatase Assay

Cortical lysate samples were prepared in TEEN buffer not supplemented with phosphatase inhibitors and 30 μg of protein were incubated at 30°C for 2 h in the presence of lambda phosphatase (New England Biolabs, Ipswich, MA, USA) according to the manufacturer protocol. A control sample (30 μg) was incubated at 30°C for 2 h in the absence of lambda phosphatase. The reaction was stopped by the addition of denaturing solution and both samples were resolved by SDS-PAGE.

### Quantum Dots Labeling and Imaging

Stargazin and AMPA receptor surface diffusion were evaluated by using quantum dots (QDs) as fluorescent probes. Low-density DIV14 cells were co-transfected with plasmids encoding Homer1C-GFP, for synapse identification, and extracellular HA-tagged stargazin. At 16 DIV, cells were stimulated with 100 μM PTX for 4 h and then incubated for 10 min at 37°C with anti-HA antibody (1:3,000) diluted in conditioned medium to label surface stargazin. After one washing step, anti-rat IgG conjugated QD655 (diluted 1:10 in PBS) was diluted in conditioned medium with BSA 2% (1/2,000) and incubated with cells for 5 min at 37°C. For quantification of endogenous AMPA receptor surface diffusion, surface GuA1 was labeled with an anti-GluA1 N-term antibody (1:1,000; Millipore, MAB2263) diluted in conditioned medium. Anti-mouse IgG conjugated QD655 (diluted 1:10 in PBS) was diluted in conditioned medium with BSA 2% (1/2,000) and incubated with cells for 5 min at 37°C.

All washes were performed in extracellular solution (ECS) containing (in mM) NaCl 145, KCl 5, Glucose 10, Hepes 10, CaCl_2_ 2 and MgCl_2_ 2), supplemented with BSA 2% at 37°C. After washing, neurons were mounted in an open chamber (K.F. Technology SRL) and imaged in ECS. Single-particle tracking was performed as in Opazo et al. (2010). Cells were imaged at 37°C on an inverted microscope (AxioObserver Z1, Carl Zeiss) equipped with a Plan Apochromat 63× oil objective (NA = 1.4). Homer1C-GFP signal was detected by using a HXP fluorescence lamp (for QDs: excitation filter 425/50 and emission filters 655/30, Chroma). Fluorescent images from QDs were obtained with an integration time of 50 ms with up to 600 consecutive frames. Signals were recorded with a digital CMOS camera (ORCA Flash 4.0, Hamamatsu). QD-labeled GluAs were imaged on randomly selected dendritic regions.

The tracking of single QDs was performed with a Matlab (Mathworks Inc., Natick, MA, USA)-based software kindly provided by Dr. Daniel Choquet (University of Bordeaux). Single QDs were identified by their diffraction limited signals and their blinking fluorescent emission. The trajectory of a QD tagged receptor could not be tracked continuously due to the random blinking events of the QDs. When the positions before and after the dark period were compatible with borders set for maximal position changes between consecutive frames and blinking rates, the subtrajectories of the same receptor were reconnected. The values were determined empirically: 2–3 pixels (0.32–0.48 μm) for maximal position change between two frames and maximal dark periods of 25 frames (1.25 s). Mean square displacement (MSD) curves were calculated for reconnected trajectories of at least 20 frames. The QDs were considered synaptic if colocalized with Homer dendritic clusters for at least five frames. Diffusion coefficients were calculated by a linear fit of the first 4–8 points of the MSD plots vs. time depending on the length of the trajectory within a certain compartment. The resolution limit for diffusion was 0.0075 μm^2^/s as determined by Groc et al. (2004), whereas the resolution precision was ~40 nm.

### Statistics

Statistical values are given as mean ± SEM or medians ± 25%/75% interval, if not stated otherwise. Statistical tests were performed using GraphPad Prism software. *T*-test or Mann-Whitney test were used to compare two conditions with data following normal and non-normal distributions, respectively. To analyze the influence of two independent variables in a continuous dependent variable, two-way analysis of variance (ANOVA) analysis was performed.

Indications of significance correspond to *p* values < 0.05 (*), *p* < 0.01 (**), *p* < 0.001 (***) and *p* < 0.0001 (****).

## Results

### Homeostatic Synaptic Downscaling Is Accompanied by Stargazin Dephosphorylation and Dendritic Stargazin Loss

**Figure 1:**
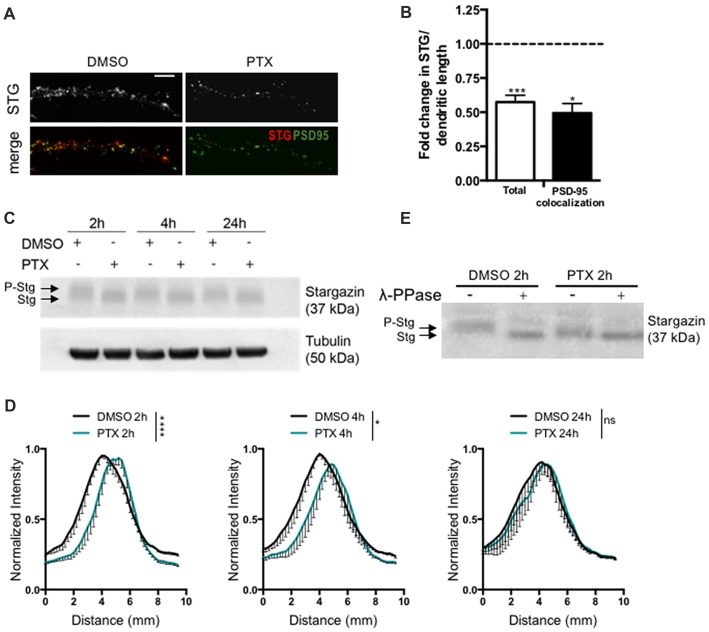
Stargazin is dephosphorylated during early stages of scaling down. **(A)** Low-density cortical neurons were treated with picrotoxin (PTX) for 2 h and the levels of total stargazin were imaged along isolated dendrites. Merge-green: PSD95; red: STG. Scale bar 5 μm. **(B)** PTX treatment decreased the intensity of stargazin puncta along the dendrites of cortical neurons (*t*-test, ***P = 0.0003) and decreased stargazin accumulation at synaptic sites (*t*-test, *P = 0.0270). *N* ≥ 20 from two independent experiments. **(C)** Representative western blot showing the effect of PTX treatment in stargazin expression. Note the differential mobility of stargazin upon PTX treatment. **(D)** Profile graphs for the stargazin western blot signals in control and PTX-treated neurons show faster stargazin mobility upon activity enhancement in cortical neurons. (*n* = 4; Two-way analysis of variance (ANOVA), ****p < 0.0001, *p < 0.05). **(E)** Neuronal lysates were incubated with λ-phosphatase before being processed for western blot. In control neurons the stargazin band was shifted to a higher mobility band, demonstrating stargazin phosphorylation in control conditions; after PTX treatment (2 h) the stargazin band was unchanged with the λ-phosphatase treatment, indicating stargazin dephosphorylation comparatively to control neurons.

To test for a potential role of stargazin in synaptic downscaling, we incubated rat cortical neurons (16 DIV) with the GABA_A_ receptor antagonist PTX (100 μM) for 2 h, 4 h or 24 h. Prolonged inhibition of GABA_A_ receptors has previously been shown to result in a decrease in the amplitude of AMPA receptor-mediated mEPSC (Turrigiano et al., 1998), and in a reduction in the synaptic accumulation of cell surface AMPA receptors (Chowdhury et al., 2018). We monitored total dendritic levels of stargazin and found that PTX treatment leads to a decrease in stargazin after 2 h, detected by immunocytochemistry (Figures 1A,B). Furthermore, stargazin co-localization with PSD-95 was significantly reduced upon PTX treatment, suggesting a depletion of stargazin at post synaptic sites during scaling down (Figures 1A,B). Stargazin can be phosphorylated at several residues in the C-terminus and previous studies demonstrated that stargazin migration in a western blot reflects the phosphorylation state of the protein (Tomita et al., 2005; Sumioka et al., 2010; Louros et al., 2014). In cortical extracts stargazin migrated heterogeneously in the western blot (Figure 1C), and treatment with λ-PPase shifted stargazin to a lower molecular weight band (Figure 1E), indicative of stargazin phosphorylation in control conditions. In extracts from PTX-incubated cells, stargazin migrated as a band shifted to lower molecular weight in denaturing SDS-PAGE conditions (Figures 1C,D; Supplementary Figure S1), suggestive of stargazin dephosphorylation in comparison to control neurons. In fact, we found that whereas λ-phosphatase treatment of control neuronal lysates compressed stargazin to a faster mobility band similar to the one found in PTX-incubated neurons, it did not change the mobility of stargazin detected in PTX-treated cultures (Figure 1E). Together, these observations indicate that stargazin levels and phosphorylation are regulated during synaptic downscaling.

### Synaptic Downscaling Is Associated With Increased Cell Surface Diffusion of Stargazin and GluA1-AMPA Receptors

**Figure 2:**
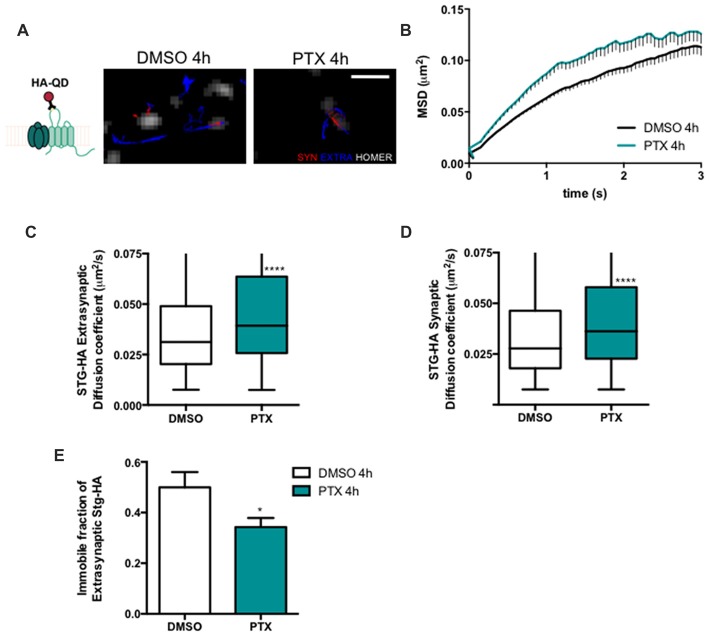
Stargazin lateral diffusion is increased during early stages of scaling down.** (A)** Scheme representing the experimental labeling strategy. Stargazin-HA (Stg-HA) particles were tracked using a primary antibody against the HA tag and Qdots-coupled to a secondary antibody. Representative trajectories of Stg-HA molecules (red—synaptic trajectories; blue—extrasynaptic trajectories), and Homer-GFP (white). Scale bar 2 μm. Cultured cortical neurons (DIV14) were transfected with stg-HA and Homer-GFP and treated with PTX at DIV16. **(B)** Mean square displacement (MSD) vs. time plot for DMSO and PTX treated neurons. Number of trajectories for DMSO 4 h = 1030 and for PTX 4 h = 520. **(C)** The median diffusion of stargazin was increased upon PTX treatment at extra-synaptic sites and **(D)** at synapses. Mann Whitney test, ****P < 0.0001. **(E)** PTX decreased the number of immobile extrasynaptic stargazin particles *t*-test, *P = 0.0347. Data was collected from ≥9 cells per condition, from two independent experiments.

Stargazin regulates AMPA receptor diffusion, and the exchange of AMPA receptors between extrasynaptic and synaptic sites (Bats et al., 2007). Phosphorylation of stargazin and its binding to PSD-95 induce the synaptic trapping of AMPA receptors diffusing in the membrane (Opazo et al., 2010). Therefore, we hypothesized that stargazin dephosphorylation associated with synaptic downscaling may affect its cell surface dynamics. To track in real time the movement of stargazin at the cell surface we transfected cortical neurons with constructs encoding Homer1C-GFP, for postsynaptic labeling, and HA-tagged stargazin; this tag is extracellular when stargazin is in the plasma membrane (Figure 2A). We took advantage of single particle tracking methods to monitor individual stargazin particles using an antibody against the extracellular stargazin HA tag, as previously described (Bats et al., 2007). We analyzed the surface explored (MSD) of stargazin in control neurons and in neurons incubated for 4 h with PTX (Figure 2A), and found that in both cases the MSD-time function is negatively curved, indicating a confined movement. Interestingly, stargazin MSD was globally increased upon neuronal activity enhancement with PTX (Figure 2B). The instantaneous diffusion coefficient of both extrasynaptic stargazin particles (Figure 2C) and of stargazin particles that co-localized with a Homer1C-GFP cluster (synaptic particles, Figure 2D) was increased, indicative of higher stargazin mobility in neurons following PTX treatment. Accordingly, the fraction of immobile particles (Figure 2E) was decreased during scaling down. In conclusion, stargazin surface diffusion is increased during the early stages of synaptic downscaling.

**Figure 3:**
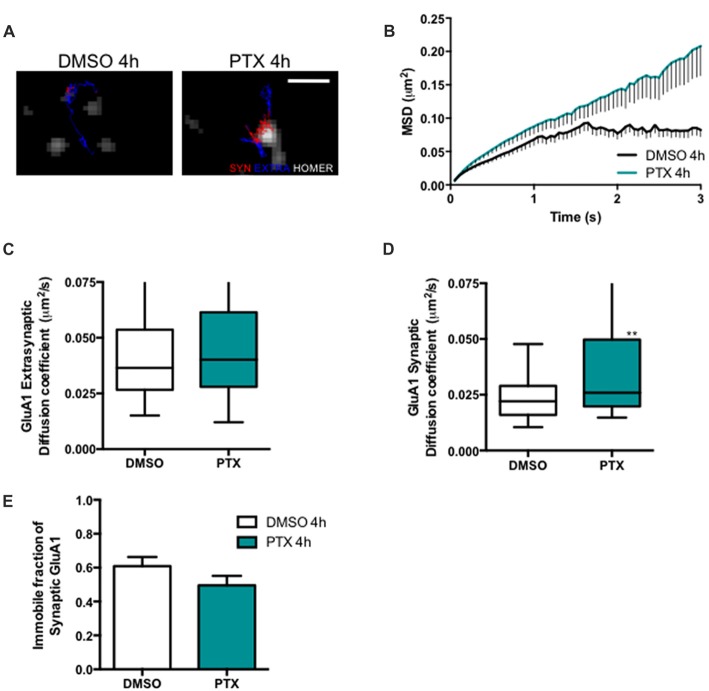
AMPA receptor synaptic mobility is increased upon down scaling. **(A)** Representative trajectories of GluA1-AMPA receptor complexes (red—synaptic trajectories; blue—extrasynaptic trajectories), and Homer-GFP (white). Scale bar 2 μm. Cultured cortical neurons (DIV14) were transfected with Homer-GFP and treated with PTX for 4 h at DIV16. **(B)** MSD vs. time plot of synaptic GluA1-AMPA receptors in control (DMSO-treated) and PTX treated neurons. Number of trajectories for DMSO 4 h = 212 and for PTX 4 h = 144. Upon PTX treatment, **(C)** the median diffusion coefficient of GluA1-AMPA receptors was not significantly changed at extrasynaptic sites (Mann Whitney test, *P* = 0.0664), but **(D)** the median diffusion coefficient of synaptic GluA1-AMPA receptors was increased (Mann Whitney test, **P < 0.01). **(E)** Percentage of immobile synaptic particles. Data was collected from ≥32 cells per condition, from five independent experiments.

Stargazin phosphorylation was shown to lead to the synaptic immobilization of AMPA receptors (Opazo et al., 2010). To test whether stargazin dephosphorylation and increased mobility associated with synaptic downscaling are accompanied by alterations in the cell surface mobility of GluA1-containing AMPA receptors, we evaluated the diffusion coefficient of endogenous GluA1-AMPA receptors using an antibody against an extracellular epitope in GluA1 (Figure 3A). The MSD (Figure 3B) and the diffusion coefficient (Figure 3D) of synaptic AMPA receptor complexes were significantly increased in PTX-treated neurons, and the fraction of immobile synaptic particles showed a tendency to be decreased (Figure 3E), but the diffusion coefficient of extrasynaptic GluA1-AMPA receptors was unchanged (Figure 3C). These data suggest that synaptic scaling down is accompanied by increased surface mobility of synaptic GluA1-AMPA receptors.

### Homeostatic Synaptic Downscaling Requires Stargazin Dephosphorylation

**Figure 4:**
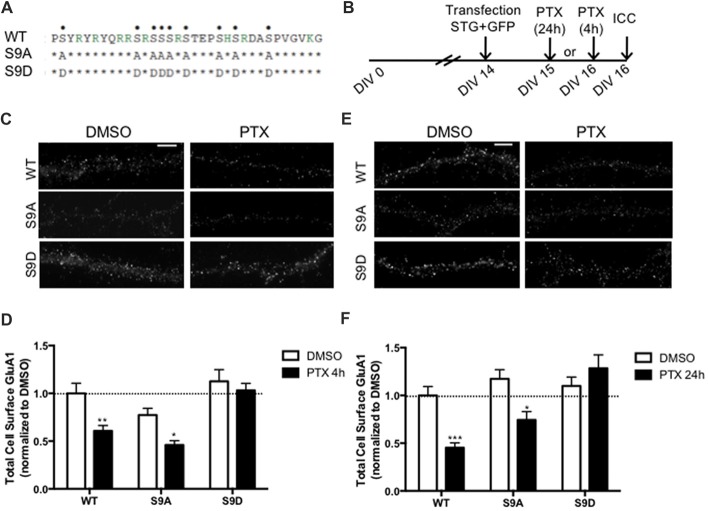
Stargazin dephosphorylation is essential to synaptic down-scaling in cortical neurons.** (A)** Amino acid sequences of the CT2 region of STG and phosphomimetic/phosphodead STG mutants. Serine residues that can be phosphorylated *in vitro* and *in vivo* are indicated with dots on the top row. S9A mutant mimicks the fully dephosphorylated state of stargazin, while the S9D mutant mimicks the fully phosphorylated form of stargazin. **(B)** Cortical neurons were transfected with stargazin (STG, WT/S9A/S9D) along with GFP at DIV 14 and treated with PTX for 4 h or 24 h. **(C)** Surface GluA1 was analyzed by immunocytochemistry after PTX treatment during 4 h. Scale bar 5 μm. **(D)** PTX induced a decrease in surface GluA1 integrated intensity in Wild-type stargazin (WT) STG and S9A-transfected neurons, but overexpression of the stargazin S9D mutant blocked PTX-induced GluA1 removal from the surface of cortical neurons. *N* ≥ 23, from three independent experiments. **(E)** Surface GluA1 was analyzed by immunocytochemistry following 24 h PTX treatment. Scale bar 5 μm. **(F)** Twenty-four hours PTX treatment induced a decrease in surface GluA1 integrated intensity in WT STG and S9A-transfected neurons, but overexpression of the S9D mutant blocked PTX-induced GluA1 removal from the surface of cortical neurons. *N* ≥ 26 from three independent experiments. Two-way ANOVA, Bonferroni multiple comparison test. In **(D,F)**, data are presented as mean ± SEM (*P < 0.05, **P < 0.01, ***P < 0.001).

Stargazin is phosphorylated by CaMKII and PKC at a stretch of serine residues at its C-terminal domain (Tomita et al., 2005), and can be dephosphorylated by PP1, a phosphatase that is activated by chronically enhancing neuronal activity, and previously shown to be critical for synaptic downscaling (Siddoway et al., 2013). At hippocampal synapses, long-term depression also requires stargazin dephosphorylation by PP1 (Tomita et al., 2005). Given our data showing that chronic neuronal activity enhancement leads do stargazin dephosphorylation, and increased cell surface mobility of stargazin and GluA1-AMPA receptors, we tested whether stargazin dephosphorylation is necessary for synaptic downscaling. Cultured cortical neurons were transfected with constructs for expression of GFP (for identification of transfected neurons) and co-transfected with WT, the phosphodead mutant of stargazin, in which the nine serine phosphorylation sites in stargazin were replaced by alanine residues (stargazin-S9A), or the phosphomimetic mutant, with aspartic acid mutations to mimic stargazin phosphorylation (stargazin-S9D; Figure 4A). Surface levels of endogenous GluA1-containing AMPA receptors were monitored by labeling with an antibody against an extracellular N-terminus epitope of GluA1, in transfected non-permeabilized cortical neurons (Figure 4B). PTX treatment for 4 h (Figures 4C,D) or for 24 h (Figures 4E,F) resulted in ~40% and ~50% loss of surface GluA1 in neurons expressing WT stargazin, respectively, and to similar decrease of cell surface synaptic GluA1 (Supplementary Figure S2). In neurons transfected with stargazin-S9A, neuronal activity enhancement still resulted in a significant decrease in the cell surface levels of total (Figures 4C–F) and synaptic (Supplementary Figure S2) GluA1, comparatively to vehicle treated neurons, indicating that expression of the non-phosphorylatable form of stargazin is permissive to the occurrence of the mechanisms that lead to downscaling. In contrast, no down-regulation was observed in neurons expressing stargazin-S9D, suggesting that stargazin dephosphorylation is required for synaptic downscaling. These results demonstrate that stargazin dephosphorylation following chronic elevation of synaptic activity underlies removal of GluA1-containing AMPA receptors from synapses.

### Binding of Stargazin to AP-2 and -3A Is Required for Synaptic Downscaling

**Figure 5:**
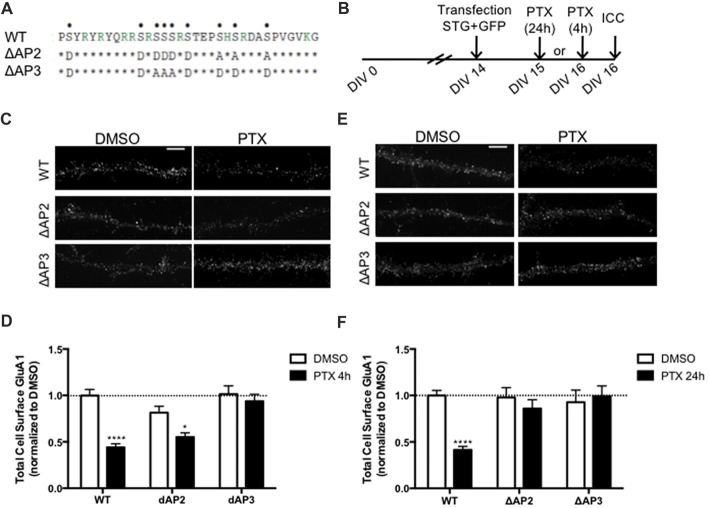
Differential regulation of scaling down by stargazin dephosphorylation. **(A)** Amino acid sequences of the CT2 region of STG and phosphomimetic/phosphodead STG mutants. Serine residues that can be phosphorylated *in vitro* and *in vivo* are indicated with dots on the top row. ΔAP2 mutant is unable to bind to the adaptor protein AP-2 thereby blocking NMDA-induced AMPA receptor endocytosis, while mutant ΔAP3 mutant is unable to bind to adaptor protein AP-3A, abrogating the late endosomal/lysosomal trafficking of AMPA receptors. **(B)** Cortical neurons were transfected with STG (WT/ΔAP2/ΔAP3) along with GFP at DIV 14 and treated with PTX for 4 h or 24 h. **(C)** Surface GluA1 was analyzed by immunocytochemistry after PTX treatment during 4 h. Scale bar 5 μm. **(D)** PTX induced a decrease in surface GluA1 integrated intensity in WT STG and ΔAP2-transfected neurons, but overexpression of the ΔAP3 mutant blocked PTX-induced GluA1 removal from the surface of cortical neurons. *N* ≥ 31, from three independent experiments. **(E)** Surface GluA1 was analyzed by immunocytochemistry following 24 h PTX treatment. Scale bar 5 μm. **(F)** Twenty-four hours PTX treatment induced a decrease in surface GluA1 integrated intensity in WT STG-transfected neurons, but overexpression of ΔAP2 or ΔAP3 mutants blocked PTX-induced GluA1 removal from the surface of cortical neurons. *N* ≥ 28, from three independent experiments. Two-way ANOVA, Bonferroni multiple comparison test. In **(D,F)**, data are presented as mean ± SEM (*P < 0.05, ****P < 0.0001).

In addition to regulating stargazin association to PSD-95, the stargazin phosphorylation state regulates AMPA receptor endocytic trafficking, through adaptor protein complexes (Matsuda et al., 2013). Dephosphorylated stargazin binds to the adaptor proteins AP-2 and AP-3A: the stargazin-S9A mutant mimicking dephosphorylated stargazin binds to AP-2 and AP-3A, but the stargazin-9D mutant that mimics constitutively phosphorylated stargazin does not bind to either of the adaptors. Furthermore, the position of phosphorylated residues within the C-terminus of stargazin determines its binding preference for AP-2 and AP-3A: a mutant of stargazin in which the first six serine residues were replaced with aspartate and the remaining with alanine binds to AP-3A but not to AP-2 (stargazin-ΔAP2), whereas serine to aspartate replacement of the first two and last four residues results in a stargazin mutant that binds to AP-2 but not to AP-3A (stargazin-ΔAP3; Figure 5A, Matsuda et al., 2013). Stargazin interaction with AP-2 and AP-3A is required for NMDA-induced reduction of cell surface AMPA receptors in hippocampal neurons. Dephosphorylation of stargazin during hippocampal LTD induces stargazin binding to AP-2 to induce endocytosis of AMPA receptors, and subsequent binding to AP-3A to deliver AMPA receptors to late endosomes and lysosomes (Matsuda et al., 2013).

Synaptic downscaling has been shown to depend on enhanced internalization of AMPA receptors ((Shepherd et al., 2006; Evers et al., 2010), but see Tatavarty et al., 2013), and results in reduced cell surface levels of GluA1-AMPA receptors (Figures 4, 5). We next asked whether stargazin association with AP-2 and AP-3A, in response to stargazin dephosphorylation, is required for synaptic scaling. To address this question we over-expressed the stargazin phosphomutants that specifically fail to interact with AP-2 (stargazin-ΔAP2) and AP-3A (stargazin-ΔAP3) in cortical neurons and then induced scaling down with PTX for 4 h or 24 h followed by detection of cell surface GluA1 before permeabilizing the plasma membrane. GluA1 immunoreactivity on the cell surface (Figures 5C,D) and at the synapse (Supplementary Figure S3) were decreased by 4 h PTX treatment in neurons expressing WT stargazin or stargazin-ΔAP2. In contrast, the expression of stargazin-ΔAP3 blocked the decrease in cell surface (Figures 5C,D) and synaptic (Supplementary Figure S3) GluA1 levels. These results indicate that overexpression of stargazin-ΔAP3, through its dominant negative effect on the stargazin–AP-3A interaction, blocked the PTX-induced decrease on cell surface AMPA receptors. The interaction of stargazin with AP-3A was shown to prevent AMPA receptor recycling to the cell surface, by promoting AMPA receptor trafficking to lysosomes (Matsuda et al., 2013). Our data indicate that during early stages of synaptic scaling the decrease on cell surface AMPA receptors is dependent on the interaction of stargazin with AP-3A, and suggest that this interaction prevents AMPA receptor recycling to the cell surface, by delivering them to late endosomes/lysosomes for degradation. Interestingly, the interaction between stargazin and AP-2 is not required for the PTX-induced decrease on cell surface GluA1, indicating that at this early stage of synaptic downscaling AMPA receptor endocytosis may be accomplished through other endocytic pathways. However, overexpression of either stargazin-ΔAP2 or stargazin-ΔAP3 blocked synaptic downscaling upon 24 h enhancement of neuronal activity with PTX (Figures 5E,F). These results suggest that in initial stages of downscaling receptor recycling to the cell surface is decreased, in a stargazin-dependent manner, whereas later in the process synaptic scaling requires both clathrin/AP-2-dependent internalization of AMPA receptors and AP-3-dependent receptor transport to late endosomes, both dependent on stargazin dephosphorylation.

## Discussion

Previous studies proposed that homeostatic adaptation of circuits in response to prolonged activity manipulation involves several molecular mechanisms. In the present study, we uncover an important role for stargazin dephosphorylation in synaptic downscaling of cortical synapses. Stargazin dephosphorylation regulates its neuronal surface mobility, increases AMPA receptor synaptic diffusion and mediates AMPA receptor endocytosis and/or targeting to late endosomes/lysosomes during different stages of synaptic homeostatic adaptation.

**Figure 6:**
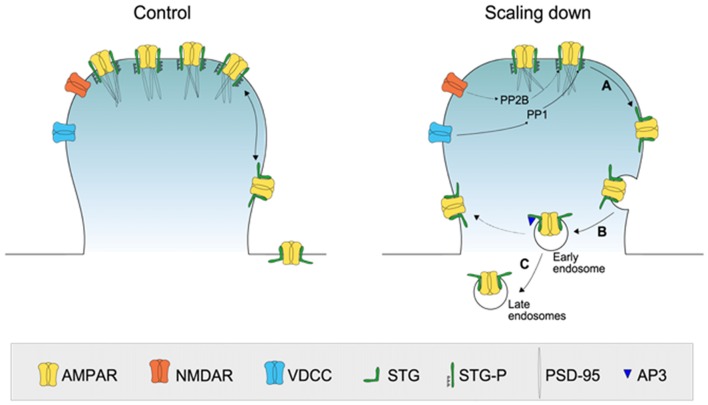
A model for the role of stargazin dephosphorylation in scaling down. At postsynaptic sites, PSD-95 binds to stargazin, which is highly phosphorylated at the C-terminus, trapping AMPA receptors at synapses. During prolonged periods of increased synaptic activity, PP1 is activated and stargazin is dephosphorylated resulting in increased lateral diffusion of stargazin and AMPA receptors at synapses **(A)**. AMPA receptors are endocytosed via clathrin/AP-2-independent or–dependent mechanisms **(B)** and directed to late endosomes through interaction with AP-3A **(C)**.

We found that scaling down is accompanied by stargazin dephosphorylation and by its decreased expression at synapses. Previous studies showed that activation of PP1 or calcineurin (PP2B) in response to NMDA treatment leads to stargazin dephosphorylation (Tomita et al., 2005), and both phosphatases participate in synaptic scaling processes (Siddoway et al., 2013; Kim and Ziff, 2014; Arendt et al., 2015; Sanderson et al., 2018). Whereas calcineurin inhibition is implicated in synaptic scaling up (Kim and Ziff, 2014; Arendt et al., 2015; Sanderson et al., 2018), activation of PP1 occurs in response to prolonged activity enhancement, as a consequence of phosphorylation of the PP1 inhibitor-2 (Siddoway et al., 2013). Considering the time-scale of PP1 activation in response to prolonged activity enhancement, it is likely that stargazin is dephosphorylated by PP1 during the early stages of synaptic downscaling (Figure 6).

Stargazin phosphorylation regulates its synaptic localization (Tomita et al., 2005; Sumioka et al., 2010), interferes with its binding to negatively-charged lipid bilayers, and enhances its binding to PSD-95 (Sumioka et al., 2010; Hafner et al., 2015). On the other hand, stargazin phosphorylation status affects its binding to the molecular adaptors AP-2 and AP-3A, and stargazin-mediated internalization and intracellular sorting of AMPA receptors (Matsuda et al., 2013). Thus, stargazin dephosphorylation upon prolonged neuronal activity enhancement may regulate both the synaptic localization of stargazin and its effect in mediating AMPA receptor endocytosis and/or intracellular sorting. It is possible that the number and position of the phosphorylated residues in stargazin result in graded interactions between stargazin and lipid bilayers, and consequently with PSD-95. Moreover, the position of phosphorylated residues in the stargazin C-terminus determines its interaction with the adaptors AP-2 and AP-3A (Matsuda et al., 2013). Graded stargazin dephosphorylation may thus work as an activity sensor providing neurons with a dynamic regulator of synaptic transmission.

Evaluation of stargazin cell surface diffusion upon 4 h of activity enhancement with PTX revealed an increase in the cell surface mobility of stargazin. These results are in accordance with TARPs binding to PSD-95-like membrane-associated guanylate kinases (MAGUKs) to stabilize the AMPA receptor/TARP complex at synapses (Chen et al., 2000; Schnell et al., 2002; Dakoji et al., 2003; Bats et al., 2007), and with the critical role of stargazin phosphorylation in the binding to PSD-95 (Sumioka et al., 2010). Stargazin dephosphorylation associated with synaptic downscaling likely promotes its dissociation from PSD-95 and increases its lateral diffusion at the neuronal membrane.

Our analysis also allowed us to determine whether synaptic scaling modulates the synaptic accumulation of AMPA receptors through changes in their lateral diffusion. Using single particle tracking methods we found that the diffusion coefficient specifically of synaptic GluA1-AMPA receptors (but not of extrasynaptic receptors) is increased upon prolonged enhancement of synaptic activity. These observations suggest that synaptic downscaling involves alterations in the lateral diffusion of AMPA receptors specifically at synapses, and not generalized changes in receptor mobility. This is in agreement with a previous study using fluorescence recovery after photobleaching (FRAP) of superecliptic phluorin (SEP)-tagged GluA2, which describes that chronic activity elevation specifically reduces the FRAP tau at synapses, without affecting SEP-GluA2 dendritic FRAP (Tatavarty et al., 2013). The enhancement in the mobility of synaptic GluA1-AMPA receptor particles could be accomplished by changing the number of synaptic binding sites within the postsynaptic density, or by changing the association of AMPA receptors to the synaptic tethers. The dephosphorylation in stargazin detected upon prolonged elevation of synaptic activity, associated with increased synaptic mobility, may lead to the increased mobility found for synaptic AMPA receptors. Previous findings indicate that expression of a mutant form of stargazin lacking the C-terminal domain increases GluA1 mobility specifically at synaptic sites, compared to neurons expressing WT stargazin (Bats et al., 2007). Upon enhancement of neuronal activity stargazin dephosphorylation at the C-terminus, by perturbing stargazin interaction with PSD-95, results in a similar change in the cell surface motility of GluA1-AMPA receptors as the overexpression of a stargazin mutant lacking the C-terminus. Another possibility is that enhanced neuronal activity leads to partial dissociation of the AMPA receptor/stargazin complex, which has been suggested to occur within milliseconds after receptor activation (Tomita et al., 2004; Morimoto-Tomita et al., 2009), and to be associated with receptor desensitization (Constals et al., 2015). In fact, glutamate binding increases AMPA receptor mobility at the synaptic plasma membrane, which is blocked by preventing the dissociation between AMPA receptors and stargazin (Constals et al., 2015). Agonist-driven dissociation of the AMPA receptor/stargazin complex may explain why the increase in the mobility of extrasynaptic stargazin is not accompanied by increased extrasynaptic AMPA receptor mobility in neurons treated with PTX. Overall, together with previous findings our data indicate that increased mobility of synaptic AMPA receptors contributes to scaling down, via changes in the anchorage of AMPA receptors to synaptic tethers resulting from stargazin dephosphorylation.

Besides regulating the synaptic anchorage of AMPA receptors, stargazin dephosphorylation is required for the decrease on cell surface AMPA receptors associated with scaling down. Overexpression of the phosphomimetic mutant of stargazin blocked the decrease in GluA1-AMPA receptors surface levels induced by 4 h or 24 h of synaptic activity enhancement, whereas the phosphodead stargazin mutant was permissive to synaptic downscaling, suggesting that stargazin dephosphorylation is necessary for synaptic downscaling to occur. In the presence of dephosphorylated stargazin additional mechanisms may operate to mediate downscaling, whereas phosphorylated stargazin completely blocks downscaling. We propose that stargazin dephosphorylation underlies removal of AMPA receptors from the cell surface during downscaling. Several studies propose that enhanced internalization of AMPA receptors drives synaptic scaling down (Shepherd et al., 2006; Evers et al., 2010), whereas others found that scaling down is not accomplished through enhanced AMPA receptor endocytosis (Tatavarty et al., 2013). We have not tested whether the endocytosis rate of AMPA receptors or stargazin is modulated during synaptic downscaling, but our data show that the decrease on cell surface GluA1-AMPA receptors triggered by prolonged (24 h) synaptic activity enhancement is blocked by overexpression of stargazin-ΔAP2, the stargazin mutant which does not bind to AP-2 and cannot promote AP-2-dependent AMPA receptor internalization. This suggests that AMPA receptor endocytosis is necessary for synaptic scaling after 24 h of activity enhancement, through a mechanism dependent on stargazin dephosphorylation. Interestingly, the stargazin-ΔAP3 mutant, which fails to bind AP-3A but binds to AP-2, also impairs the cell surface decrease on GluA1-AMPA receptors upon activity enhancement. This indicates that scaling down is accomplished via decreased AMPA receptor recycling to the cell surface, and increased targeting to the late endosomes/lysosomes. Of note, in the case of LTD, receptor sorting to the lysosome rather than to recycling endosomes is also key in determining the extent of synaptic depression upon LTD induction (Fernández-Monreal et al., 2012), and stargazin plays a crucial role in LTD by regulating AMPA receptor endocytosis and transport from early endosomes to late endosomes/lysosomes (Matsuda et al., 2013).

Remarkably, synaptic activity elevation for 4 h results in a significant decrease on the cell surface levels of GluA1-AMPA receptors, which does not require the interaction of stargazin with AP-2 but does require association of stargazin with AP-3A. This observation suggests that during the initial stages of synaptic scaling there are mechanisms independent of the AP-2 association with stargazin that drive AMPA receptor internalization, but that stargazin association with AP-3A is required to transport intracellular AMPA receptors from early to late endosomes, diverging them from recycling pathways. The internalization mechanisms could depend on other proteins that drive AMPA receptor endocytosis such as Arc (Shepherd et al., 2006), or on clathrin-independent endocytosis pathways. Importantly, clathrin-independent endocytosis of AMPA receptors has been described, and shown to participate in constitutive AMPA receptor internalization and in homeostatic AMPA receptor downscaling (Glebov et al., 2015). We propose that in the initial stages of synaptic downscaling stargazin dephosphorylation is responsible for increased AMPA receptor synaptic diffusion followed by AMPA receptor internalization, through stargazin-independent mechanisms; internalized AMPA receptors are driven from early to late endosomes through stargazin association with AP-3A. This sorting decision depends on stargazin dephosphorylation and prevents receptors from recycling back to synapses, thereby resulting in synaptic downscaling.

In fact, the phosphoproteome analysis of postsynaptic samples from the forebrain of animals in the wake and sleep phases revealed a phosphopeptide in stargazin which is hyperphosphorylated in the wake phase compared to the sleep phase (Diering et al., 2017). The peptide corresponds to the region of stargazin which dephosphorylation is required for binding to AP-3A. Since synaptic downscaling occurs during sleep, this observation is in agreement with our study showing stargazin dephosphorylation upon enhanced neuronal activity. It further suggests that the interaction between stargazin and AP-3A may occur during sleep *in vivo*, leading to reduced AMPA receptor recycling and to AMPA receptor delivery to the late endosomes/lysosomes, and synaptic downscaling.

Surprisingly, despite the well-established role of AP-3 in sorting membrane proteins to the lysosomal pathway for degradation (Bonifacino and Traub, 2003), upregulation of the AP-3 subunit μ3A induced by prolonged activity blockade reroutes AMPA receptors to the recycling pathway, a process which enhances synaptic strength (Steinmetz et al., 2016). Treatment of cortical neurons with TTX reduces the association of μ3 with lysosomes, and increases its association with recycling and early endosomes (Steinmetz et al., 2016). The μ3A subunit acts independently of the full AP-3 complex during synaptic upscaling to recruit AMPA receptors to recycling endosomes, where they can then be recruited to the plasma membrane in a second trafficking step, mediated by GRIP1. This action of the μ3A subunit in synaptic upscaling, which is independent of the full AP-3 complex, is in stark contrast with the role of AP-3A in binding to dephosphorylated stargazin and promoting AMPA receptor sorting to late endosomes and lysosomes during LTD (Matsuda et al., 2013) and synaptic downscaling. It is possible that AP-3 competes with the μ3A subunit for binding to stargazin and promoting AMPA receptor routing to late endosomes and lysosomes for degradation.

Taken together, our data show that stargazin dephosphorylation during synaptic downscaling is associated with increased surface mobility of stargazin and synaptic GluA1. Furthermore, these data identify stargazin dephosphorylation, and stargazin binding to AP-3A as necessary for the decrease on cell surface GluA1-AMPA receptors during synaptic downscaling. Our results suggest a dual role for stargazin dephosphorylation in displacing synaptic AMPA receptors during downscaling, through increased AMPA receptor synaptic mobility and sorting of intracellular AMPA receptors to degradation pathways.

## Author Contributions

SL and GC performed experiments and analyzed data. SL, GC and AC designed experiments and wrote the article.

## Conflict of Interest Statement

The authors declare that the research was conducted in the absence of any commercial or financial relationships that could be construed as a potential conflict of interest.
