# Supplementary material for: Stargazin Dephosphorylation Mediates Homeostatic Synaptic Downscaling of Excitatory Synapses
Source: Front Mol Neurosci. 2018 Sep 13;11:328. doi: 10.3389/fnmol.2018.00328 (PMC6146028; doi:10.3389/fnmol.2018.00328)
Supplement: Supplementary file 1 [file Presentation_1.pdf]

Stargazin dephosphoryation mediates homeostatic synaptic downscaling of excitatory synapses

Susana R. Louros[1,*], Gladys L. Caldeira[1], Ana Luísa Carvalho[1,2]

[1]CNC-Center for Neuroscience and Cell Biology, University of Coimbra, Coimbra, Portugal
[2]Department of Life Sciences, University of Coimbra, Coimbra, Portugal
[*]Present address: Centre for Discovery Brain Sciences/ Patrick Wild Centre
University of Edinburgh, Hugh Robson Building, Edinburgh, UK

Supplementary Information

Figure S1.

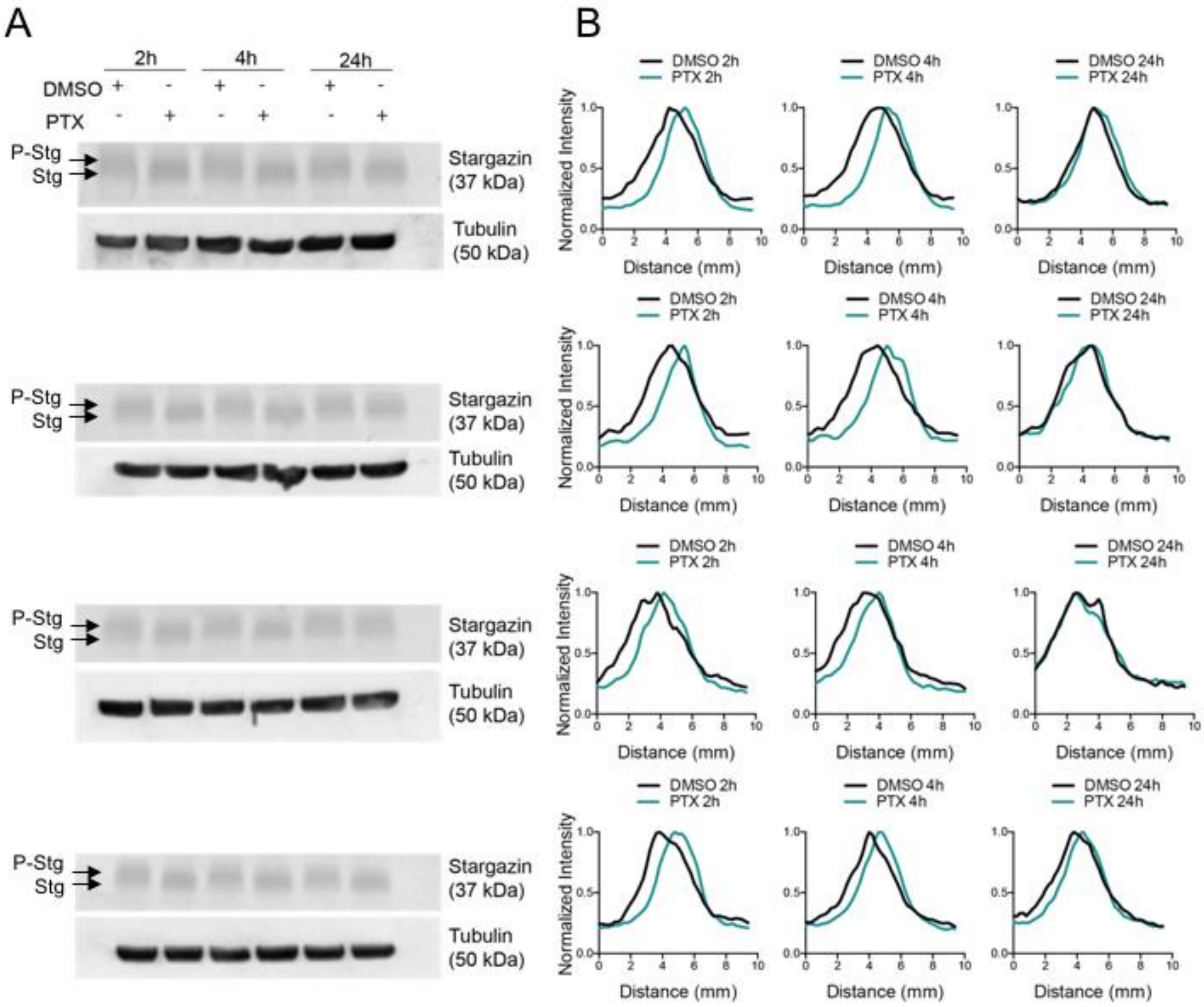

Fig. S1 – Stargazin profile in control and PTX-treated cortical neurons.

(A) Western blots showing the effect of PTX treatment in stargazin expression. Note the differential mobility of stargazing upon PTX treatment. (B) Profile graphs for the stargazin western blot signals in control and PTX-treated neurons show faster stargazin mobility upon activity enhancement in cortical neurons.

Figure S2.

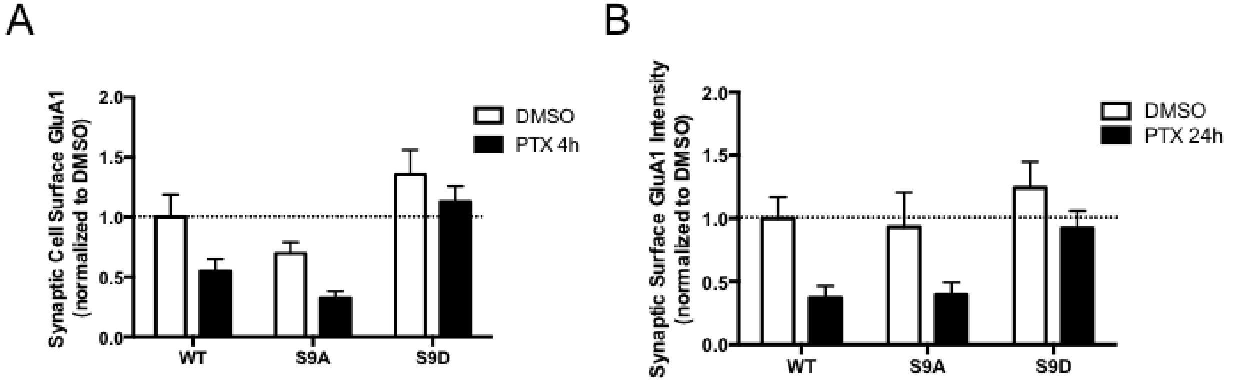

Fig. S2 – Stargazin dephosphorylation is essential for the scaling down of synaptic AMPA receptors.

A) Synaptic surface GluA1 levels in cortical neurons transfected with stargazin (STG, WT/S9A/S9D) at DIV 14 and treated with PTX for 4h at DIV 16. N≥16, from two independent experiments. The synaptic GluA1 localization was assessed by evaluation of the colocalization of the GluA1 signal with the synaptic marker PSD-95.
B) Synaptic surface GluA1 levels in cortical neurons transfected with stargazin (STG, WT/S9A/S9D) at DIV 14 and treated with PTX for 24h at DIV 16. N≥7, from one experiment. The synaptic GluA1 localization was assessed by evaluation of the colocalization of the GluA1 signal with the synaptic marker VGlut1.
In (A) and (B) data are presented as mean ± SEM.

Figure S3.

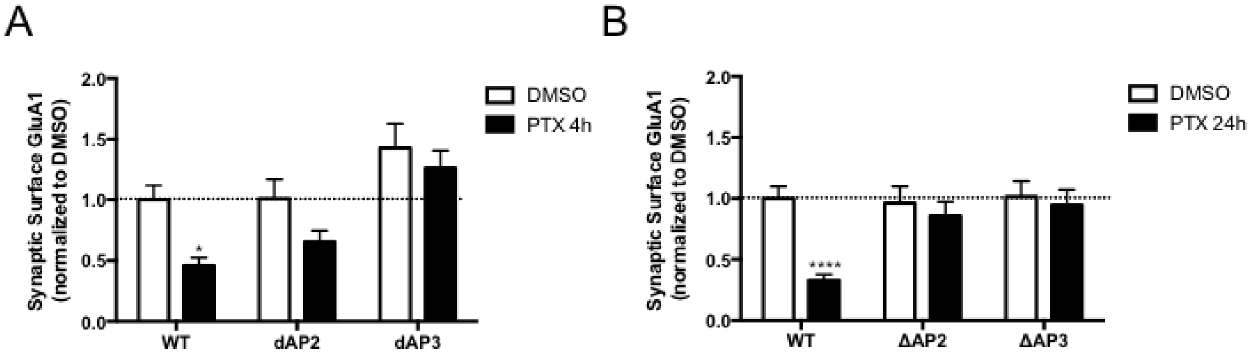

Fig. S3 – Differential regulation of synaptic AMPA receptor scaling down by stargazin dephosphorylation.

A) Synaptic surface GluA1 levels in cortical neurons transfected with stargazin (STG, WT/S9A/S9D) at DIV 14 and treated with PTX for 4h at DIV 16. N≥31, from three independent experiments. The synaptic GluA1 localization was assessed by evaluation of the colocalization of the GluA1 signal with the synaptic marker PSD-95.

B) Synaptic surface GluA1 levels in cortical neurons transfected with stargazin (STG, WT/S9A/S9D) at DIV 14 and treated with PTX for 24h at DIV 16. N≥27, from three experiment. The synaptic GluA1 localization was assessed by evaluation of the colocalization of the GluA1 signal with the synaptic marker VGlut1.

In (A) and (B) data are presented as mean ± SEM. *$p<0.05$, ****$p<0.0001$.
